# Infantile hypophosphatasia in a Chinese patient: identification and characterization of novel compound heterozygous ALPL mutations

**DOI:** 10.1038/s41439-025-00334-y

**Published:** 2025-12-06

**Authors:** Wenjuan Li, Shujing Zeng, Jun Jiang, Bin Liu

**Affiliations:** 1 Pediatrics Department of Southwest Medical University Affiliated Hospital, Luzhou, China; 2 Sichuan Clinical Research Center for Birth Defects, Luzhou, China; 3 Thyroid Surgery Department of the Affiliated Hospital of Southwest Medical University, Luzhou, China

**Keywords:** Medical genetics, Endocrine system and metabolic diseases

## Abstract

Here we report a Chinese infant with hypophosphatasia (HPP) carrying alkaline phosphatase (ALPL) gene mutations. Genetic analysis of the patient’s ALPL gene revealed a maternally inherited canonical splice-site variant (c.997+1G>T; pathogenic; PVS1 + PM2 + PP4) and a paternally inherited missense variant (c.1405C>T, p.His469Tyr; reclassified as pathogenic; PP4 + PM2 + PP3). Both variants have previously been reported in gnomAD with very low frequency in Chinese infants.

Hypophosphatasia (HPP) is an uncommon genetic condition resulting from a mutation in the ALPL gene, which produces tissue-nonspecific alkaline phosphatase (TNSALP)^1^. The lack of TNSALP leads to the extracellular buildup of pyrophosphate, a potent inhibitor of mineralization, causing impaired teeth and bones. The prevalence of HPP was calculated at 1 in 300,000 for severe cases and 1 in 6370 for mild cases in Europe, respectively^2^. Sporadic and monoploid cases are quite rare^3^. Clinical types may be categorically differentiated depending on the age of onset and the presence or absence of skeletal symptoms: perinatal deadly, perinatal benign, infantile, childhood, adult and dental HPP^4^. Detailed reports characterizing novel pathogenic variants in Chinese infants are limited. We provide a comprehensive clinical and genetic assessment of a Chinese newborn diagnosed with severe infantile HPP.

The patient was a 4-month-16-day-old boy who was brought to the hospital for intermittent vomiting lasting 2 months and a cough with fever for 1 week. The patient has a history of recurrent respiratory infections in the past 2 months. The child is the second offspring of a second pregnancy, born at 41 weeks via spontaneous vaginal delivery, with a birth weight of 3250 g. There were no asphyxia events at birth. The child has been on mixed feeding and is still unable to hold up his head. Blood calcium fluctuated between 2.62 and 3.67 mmol/l across five tests, and blood phosphorus ranged from 1.03 to 1.7 mmol/l over the same period. Thyroid function tests were standard, and low parathyroid hormone levels were found. Bone metabolism tests revealed an alkaline phosphatase (ALP) level of 17.4 U/l and a phosphate (P) level of 1.24 mmol/l, with all other parameters within the normal range. Radiographic examination revealed reduced bone mineral density in both hands, wrists, bilateral femurs, tibiae and fibulae, with sparse trabeculae and uneven bone density. The distal ends of the ulnae and radii bilaterally, the proximal ends of the left ulna and radius, and the metaphyses of both femurs, tibiae and fibulae were enlarged, exhibiting cup-shaped or brush-like appearances. Some epiphyseal ossification centers were delayed, but all joints in the hands, wrists, hips, knees and ankles were properly aligned, showing no abnormal findings.

Following the acquisition of informed permission from the patient’s guardians, peripheral blood specimens were procured from the patient and their parents and sent to Beijing Mech-Med Medical Laboratory for genetic analysis. Although infantile HPP was suspected clinically, the overlapping features with other skeletal dysplasias and recurrent respiratory infections necessitated a broad differential. Therefore, trio-based whole-exome sequencing was performed to exclude alternative diagnoses and identify potential novel genes simultaneously. Pathogenicity interpretation followed American College of Medical Genetics and Genomics (ACMG)/Association for Molecular Pathology (AMP) 2015 guidelines^5^.

Whole-exome sequencing of the proband identified two unique compound heterozygous mutations in the ALPL gene (Table 1). The maternally inherited c.997+1G>T splice-site variation (chr1:21900293; NM_000478.6, intron 9) was classed as pathogenic according to ACMG criteria (PVS1: canonical splice site disruption; PM2: ultrarare in gnomAD (Minor Allele Frequency (MAF) 1.239 × 10^−6^)). The paternally inherited c.1405C>T (p.His469Tyr) missense variant (chr1:21903971; NM_000478.6, exon 12; PM2: ultrarare in gnomAD (MAF 6.232 × 10^−7^)) was initially designated uncertain significance. The His469Tyr is currently also registered in gnomAD (MAF 6.232 × 10^−7^), supporting the PM2 criterion. Bioinformatics prediction using REVEL, which assesses variant pathogenicity, indicates a deleterious effect for this variant, supporting the PP3 criterion, and other prediction software, including SIFT, PolyPhen-2, MutationTaster and GERP, resulted in deleterious predictions for this variant. The mother was heterozygous for c.997+1G>T only, the father was heterozygous for c.1405C>T only, and the proband compound was heterozygous for both mutations, confirming autosomal recessive inheritance by segregation analysis. Homology modeling (Swiss-Model; Fig. 1) demonstrated that wild-type His469 (positively charged polar residue) forms no polar bonds with adjacent residues. Mutation to tyrosine (uncharged polar) introduced a novel hydrogen bond (3.5 Å) between Tyr469-OH and Asn47, altering local charge distribution and intramolecular interactions. These changes are predicted to destabilize protein folding and impair catalytic function, consistent with a loss-of-function mechanism.

**Fig. 1:**
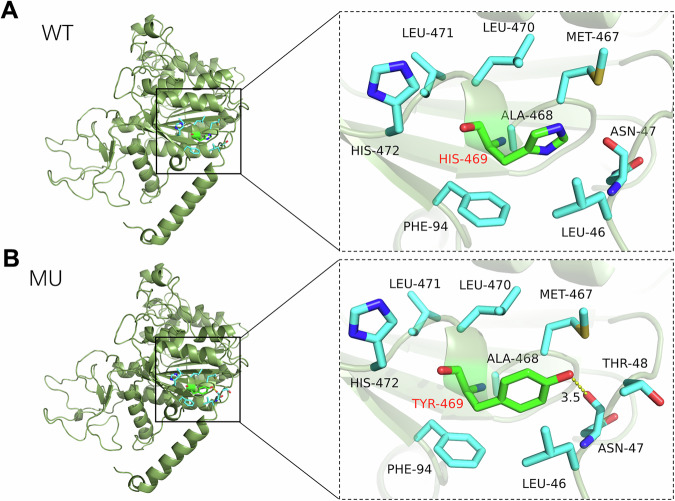
Structural impact of the p.His469Tyr variant on TNSALP. **A**, **B**, Homology modeling (Swiss-Model) comparing wild-type (**A**) and mutant (**B**) structures at residue 469. Wild-type His469 (positively charged, polar) forms no polar bonds with adjacent residues. Substitution to tyrosine (uncharged, polar) introduces a novel hydrogen bond (3.5 Å, dashed yellow line) between the Tyr469 hydroxyl group and ASN-47. The aberrant hydrogen bond distorts the active-site geometry, as quantified by the electrostatic surface potential (right).

**Table 1:** Identification and characterization of compound heterozygous variant of ALPL in the proband.

Field	Mutation 1 (c.997+1G>T)	Mutation 2 (c.1405C>T)
Gene	ALPL	ALPL
Chromosomal location	chr1:21900293	chr1:21903971
Transcriptexon	NM_000478.6; intron 9	NM_000478.6; exon 12
Nucleotide	c.997+1G>T (P.?)	c.1405C>T (p.His469Tyr)
Zygosity	het	het
Normal frequency	1.239 × 10^−6^	6.232 × 10^−7^
Pathogenicity analysis of ACMG	Pathogenic	Uncertain
ACMG/AMP evidence codes	PVS1, PM2, PP4	PP4, PM2, PP3,
Disease/phenotype (genetic pattern)	Childhood HPP	Childhood HPP
	Infantile HPP	Infantile HPP
	Adult HPP	Adult HPP
Source of variation	Mother	Father

P.?: Unknown significance

The identification of compound heterozygous variants c.997+1G>T and c.1405C>T (p.His469Tyr) in the ALPL gene provides critical insights into TNSALP dysfunction mechanisms. Splice-site variant c.997+1G>T disrupts the canonical GT donor site in intron 9, predicted to cause exon skipping or intron retention. This aligns with studies showing that >80% of splice-site variants in ALPL lead to frameshifts or premature stop codons, abolishing TNSALP activity^6^. The p.His469Tyr variation, previously categorized as a variant of unknown significance, illustrates how structural bioinformatics clarifies variant ambiguity. Functional validation further upgraded the classification from variant of unknown significance to pathogenic according to ACMG PS3. Our homology modeling reveals that substituting positively charged histidine (position 469) with uncharged tyrosine alters the electrostatic potential in a conserved catalytic domain. The novel hydrogen bond between Tyr469 and Asn47 distorts the active site architecture, impairing substrate binding, establishing position 469 as a functional hotspot. Importantly, the proband’s markedly low ALP level (17.4 U/l) provides in vivo validation of these computational predictions, fulfilling the ACMG functional criterion for pathogenicity reclassification^5^.

This case reinforces the paradigm that biallelic loss-of-function variants drive severe infantile HPP. The proband’s compound heterozygosity, combining a truncating splice variant (c.997+1G>T) and a destabilizing missense change (p.His469Tyr), resulted in residual TNSALP activity. The 50% mortality risk in untreated infantile HPP further emphasizes the urgency of early genetic diagnosis, particularly given the therapeutic window for enzyme replacement therapy before irreversible skeletal damage occurs^7,8^. Prior studies report more than 400 pathogenic ALPL variants in patients with HPP^6^. The first instance of HPP was documented in 1948, and the mutation of the ALPL gene was identified in 1988^9^. HPP is inherited in an autosomal pattern, primarily recessive. The wide spectrum of clinical manifestations and inheritance modes complicates prompt diagnosis and poses challenges for genetic counseling^10^. Patients with perinatal or infantile HPP exhibit elevated morbidity and death throughout the first 5 years of life. Chest deformity, respiratory discomfort, failure to thrive and increased calcium levels were seen in almost 70% of patients^11^. Our decision to perform trio whole-exome sequencing instead of targeted ALPL screening was driven by the need to exclude disorders with overlapping phenotypes, such as osteogenesis imperfecta or primary ciliary dyskinesia. This unbiased approach is particularly cost-effective in resource-limited settings.

This study reports novel compound heterozygous variants in the ALPL gene (c.997+1G>T and c.1405C>T/p.His469Tyr) underlying severe infantile HPP in a Chinese patient. Our findings expand the mutational spectrum of ALPL and highlight the critical role of functional validation in reclassifying variants of uncertain significance. This case enhances genotype–phenotype understanding of infantile HPP in Chinese populations and provides essential data for improving molecular diagnostic and genetic counseling in resource-limited settings.

## HGV Database

The relevant data from this Data Report are hosted at the Human Genome Variation Database at 10.6084/m9.figshare.hgv.358710.6084/m9.figshare.hgv.3590.
